# Serum Caveolin-1 as a Novel Biomarker in Idiopathic Pulmonary Artery Hypertension

**DOI:** 10.1155/2015/173970

**Published:** 2015-10-11

**Authors:** Kuo-Yang Wang, Mey-Fann Lee, Hung-Chin Ho, Kae-Woei Liang, Chia-Chi Liu, Wan-Jane Tsai, Wei-Wen Lin

**Affiliations:** ^1^Cardiovascular Center, Taichung Veterans General Hospital, Taichung, Taiwan; ^2^School of Medicine, Chung Shan Medical University, Taichung, Taiwan; ^3^School of Medicine, China Medical University, Taichung, Taiwan; ^4^Department of Education and Research, Taichung Veterans General Hospital, Taichung, Taiwan; ^5^Department of Life Science, Tunghai University, Taichung, Taiwan; ^6^Institute of Clinical Medicine, Cardiovascular Research Center, Taiwan; ^7^Department of Medicine, National Yang Ming University School of Medicine, Taipei, Taiwan; ^8^Department of Biomedical Engineering & Environmental Sciences, National Tsing Hua University, Hsinchu, Taiwan

## Abstract

Pulmonary arterial hypertension (PAH) is a rare disease but with significant morbidity and high mortality. There is no specific way to diagnose PAH. Thus, an easy used with good sensitivity and specificity biomarker of PAH is highly desirable to aid in the screening, diagnosis, and follow-up. Caveolin-1 (Cav1) is the structural protein of caveolae and is highly expressed in type I pneumocytes. Lungs tissues from idiopathic PAH (IPAH) patients showed decreased expression of Cav1 in vascular endothelial cells. Therefore, we developed a direct sandwich immunoassay for the determination of Cav1 in IAPH patient's serum. The result disclosed serum Cav1 level was significantly lower in IPAH than control groups. Using serum Cav1, 17.17 pg/mL as a cutoff value, the sensitivity was 0.59 and the specificity was 1.0. There were two major findings in our results. First, serum Cav1 might be a novel biomarker in the diagnosis of IPAH with fare sensitivity and good specificity. Second, Cav1 might be used to make differential diagnosis between COPD-PH and IPAH group.

## 1. Introduction

Pulmonary arterial hypertension (PAH) is a rare disease but with significant morbidity and high mortality. Annual incidence is 1-2 cases per million people in the USA and it is 2–4 times as common in women as in men [1, 2]. In untreated patients, the median survival rate is only 2.8 years, and the 5-year survival rate is 34% [3]. There is no specific way to diagnose PAH. According to the American and European clinical practice guidelines [4–6], the diagnosis involves sequences of steps and requires several invasive and noninvasive examinations. Well experienced specialists are needed to interpret the results and manage these patients [7, 8]. Thus, sensitive and specific biomarkers of PAH are highly desirable to aid in the screening, diagnosis, and follow-up.

Previous studies have suggested that atrial natriuretic peptide (ANP), N-terminal probrain natriuretic peptide (NT-proBNP), troponin, and uric acid are potential biomarkers for PAH [9–12]. However, these are not specific biomarkers of the pathology changed of the pulmonary artery hypertension. Endothelial cell dysfunction, proliferation without apoptosis, and vasoconstriction may play important roles in PAH; therefore vascular bed may be a good source of new biomarkers [13–15].

Caveolae are 50–100 nm vesicular invaginations of the cell plasma membrane and caveolin-1 (Cav1) is the structural protein of caveolae and is highly expressed in adipocytes, endothelial cells, and type I pneumocytes. Cav1^−/−^ mice exhibit pulmonary hypertension and right ventricle hypertrophy [16–18]. In monocrotaline-induced PH rat models, Cav1 deficiency is seen in lung tissue [19]. Lungs tissues from idiopathic PAH (IPAH) patients decreased expression of Cav1 in vascular endothelial cells and also decreased in the total lung lysate [20, 21]. Furthermore, Cav1 can be secreted into serum and be detected [22]. These results suggested that Cav1 may play an important role in the pathogenesis of PAH and serum Cav1 level may be a good biomarker for diagnosis [23, 24].

## 2. Materials and Methods

In the study, age matched patients with normal left ventricle function were divided into 3 groups. In Group (1) IPAH patients (*n* = 21), definite diagnosis was made according to European Society of Cardiology (ECS) [4] and American Heart Association (AHA/ACC) [5] guideline, inclusion criteria including mean pulmonary artery pressure (mPAP) ≥ 25 mmHg, pulmonary wedge pressure less or equal to 15 mmHg, and pulmonary vascular resistance over 3 Wood units measured by right heart catheterization. In Group (2) chronic obstructive pulmonary disease with pulmonary hypertension (COPD-PH) patients (*n* = 22), COPD were diagnosed by pulmonologist and estimated mean PAP ≥ 25 mmHg by echocardiography. Group (3) (non-PAH group) includes healthy volunteers (*N* = 26) with mPAP less than 15 mmHg measured by echocardiography.

Demographic data and clinical features of patients included in this study were summarized in Table 1. According to Tahir et al. reports [23, 24], we developed a direct sandwich immunoassay for the determination serum Cav1 level from participants. Serum hsCRP, NT-proBNP, and BMPR2 levels were also measured by commercial ELISA kits. This study was approved by Local Ethical Committee in Taichung Veterans General Hospital, Taichung, Taiwan (number CE12022). Written informed consent was provided to all participants.

### 2.1. Protocol for Serum Cav1 Assay

**Figure 1:**
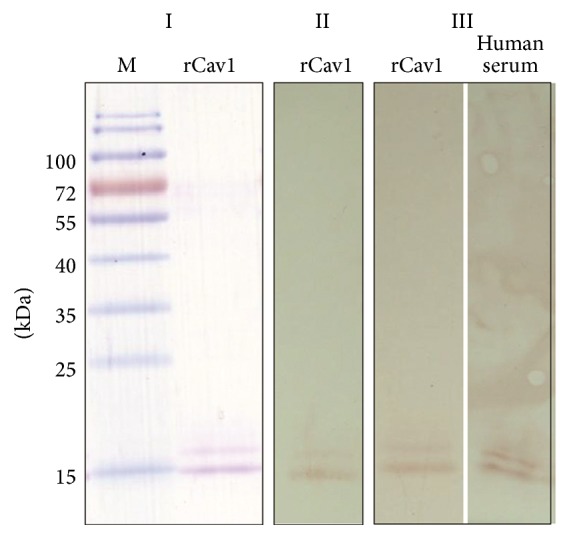
Specificity of capture and detection antibodies to rCav-1 and native Cav1 of human serum. Recombinant Cav1 protein was purified using affinity chromatography and analyzed by SDS-PAGE (I) and immunoblotting (II and III). In panel I, the rCav-1 protein migrated as a single band and displayed >95% purity by Coomassie blue staining. The binding specificity of the capture and detection antibodies to rCav-1 and human serum was demonstrated and showed in panel II and panel III, respectively. Numbers on the left indicate sizes of protein markers (lane M).

Two commercial affinity purified monoclonal mouse Cav1 antibodies and polyclonal rabbit Cav1 antibodies were chosen for a direct sandwich ELISA. The capture Cav1 antibody used was generated from human recombinant Cav1 (R&D systems), and the detection antibody was HRP-conjugated rabbit polyclonal antibody raised against a peptide mapping at the NH_2_ terminus of human Cav1 (Santa Cruz Biotechnology). Costar microplate wells were coated with 100 *μ*L of Cav1 antibody (2.5 mg/L) in PBS (pH 7.4) and incubated overnight at 4°C. The wells were then blocked with 300 *μ*L of PBS containing 0.5% BSA and 0.05% v/v Tween 20 for 2 hours at room temperature and were washed three times with PBS containing 0.5% v/v Tween 20 (PBST). Serum samples, calibrators, and controls were added (100 *μ*L) to the wells and incubated overnight at 4°C. The wells were washed three times with 400 *μ*L of PBST and 100 *μ*L of HRP-conjugated Cav1 (Santa Cruz Biotechnology) antibody diluted 1 : 2000 in PBST. After incubation for 2 hr at room temperature, the wells were washed three times with PBST, and 100 *μ*L of 3,3′,5,5′-tetramethylbenzidine substrate solution (Clinical Science Products, Inc.) was added and incubated for 30 min at room temperature. The reaction was stopped by adding 100 *μ*L of 1 N H_3_PO_4_, and the absorbance was read at 450 nm with a microplate reader (TECAN, Grödig, Austria). Serum Cav1 levels were measured using lab-made recombinant pQE30-Cav 1 (a 101-amino acid region of Cav1 gene from GenBank accession number NM001753) as a standard. A linear standard curve was constructed using a concentration range (12.19–780 pg/mL) of recombinant Cav1 in a parallel ELISA. The levels of Cav1 in sera of 21 IPAH patients, 22 COPD-PH patients, and 26 healthy controls by the in-house ELISA were then measured. The limit of detection of the sandwich ELISA was 12.19 pg/mL. Any value below the detective limitation of the assay referred to zero. In Figure 1, Western blot data using capture and detection antibodies that react to recombinant Cav1 proteins was showed.

### 2.2. Enzyme-Linked Immunosorbent Assay for HsCRP, NT-proBNP, and BMPR2 Detection

The serum levels of high-sensitivity C-reactive protein (hsCRP), NT-proBNP, and bone morphogenetic protein type II receptor (BMPR2) were measured with enzyme-linked immunosorbent assay (ELISA) kits, hsCRP (Cell Biolabs, Inc., San Diego, CA), NT-proBNP (Invitrogen Corporation, Camarillo, CA), and BMPR2 (MyBioSource, San Diego, CA) according to the manual.

### 2.3. Statistical Analysis

All data were expressed as mean ± standard deviation. One-way analysis of variance (ANOVA) was used to compare continuous variables among different groups and Mann-Whitney *U* test was used to compare variables between groups. Optimal thresholds for survival analysis were identified using Receiver-Operated Characteristics (ROC) analysis. Statistical analysis was performed using SPSS 18 (SPSS; Chicago, IL, USA).

## 3. Results

In Table 1, patients in IPAH group were younger than non-PAH volunteer and COPD-PH group, but not significant (*p* = 0.16). There were more female patients (*n* = 14) than male patients (*n* = 6) in IPAH group. The systolic blood pressure was significantly lower than IPAH groups, which may result from right heart failure (*p* = 0.002). The PA pressure was significantly different between IPAH, COPD-PH, and non-PAH group (*p* < 0.005).

**Figure 2:**
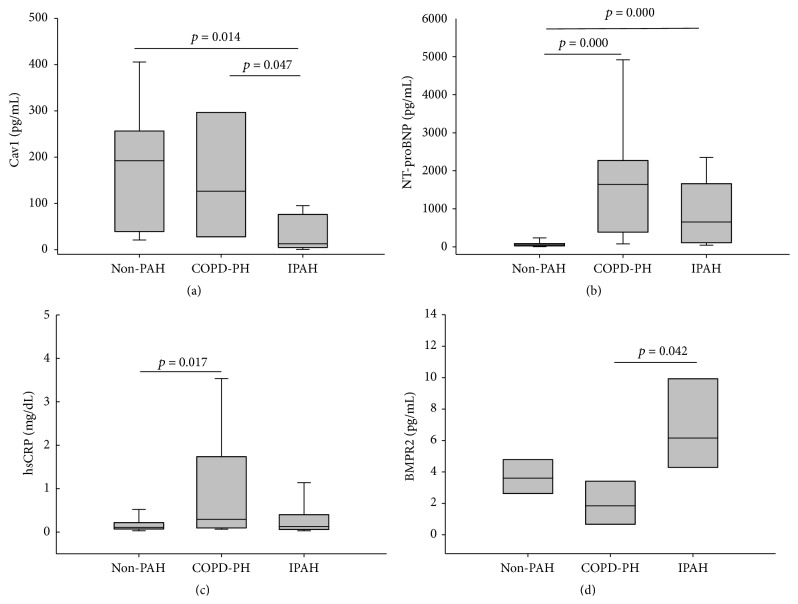
Serum biomarker levels in PAH patients and control subjects. PAH: pulmonary artery hypertension, COPD-PH: chronic obstructive pulmonary disease with pulmonary hypertension, and IPAH: idiopathic PAH.

In Figure 2(a), serum Cav1 level was significantly low in IPAH compared to non-PAH and COPD-PH group (76.45 ± 32.41 versus 140.75 ± 59.72 pg/mL and 173.57 ± 42.75 pg/mL; *p* = 0.014 and *p* = 0.047). But there was no significant difference between non-PAH and COPD-PH. NT-proBNP (Figure 2(b)) was significantly higher in IPAH and COPD-PH than normal group (933.59 ± 210.09 and 1806.38 ± 474.07 versus 83.436 ± 22.33 pg/mL, both *p* < 0.05), but there is no difference between IPAH and COPD-PH groups. hsCRP (Figure 2(c)) was significantly higher in COPD-PH group than non-PAH group (1.02 ± 0.32 versus 0.20 ± 0.04 mg/mL, *p* = 0.017), but there is no difference between COPD-PH and IPAH group (1.02 ± 0.32 versus 0.37 ± 0.15 mg/mL, *p* > 0.5). BMPR2 (Figure 2(d)) was higher in IPAH group than COPD-PH group (22.35 ± 15.60 versus 2.57 ± 0.99 pg/mL, *p* = 0.019), but there is no significant difference between COPD-PH and non-PAH group (2.57 ± 0.99 versus 6.41 ± 3.27 pg/mL, *p* > 0.5).

**Figure 3:**
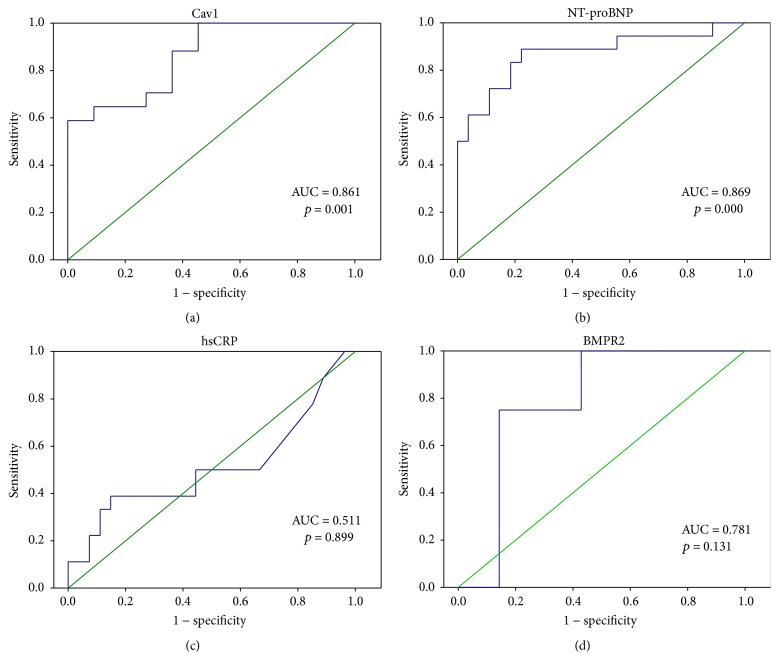
Receiver operator curve analysis of Cav1 and other biomarkers in idiopathic pulmonary artery hypertension (IPAH) patients.

In IPAH patients, using serum Cav1, 17.17 pg/mL as a cutoff value (Table 2), the sensitivity was 0.59, the specificity was 1.0, and area under ROC curve was 0.816 (Figure 3(a)). Using NT-proBNP, 89.25 pg/mL as a cutoff value, the sensitivity was 0.889, the specificity was 0.778, and area under ROC curve was 0.89 (Figure 3(b)). Using hsCRP, 0.27 mg/dL as a cutoff value, the sensitivity was 0.39, the specificity was 0.85, and area under ROC curve was 0.89 (Figure 3(c)). Using BMPR2, 3.71 pg/mL as a cutoff value, the sensitivity was 1.00, the specificity was 0.43, and area under ROC curve was 0.78 (Figure 3(d)). Linear regression analysis between Cav1 and 6 min walk test, PAP, and PVR was done but did not show good correlation. Data were not shown here.

## 4. Discussion

There were two major findings in our results. First, serum Cav1 might be a novel biomarker in the diagnosis of IPAH with fare sensitivity and good specificity. Second, Cav1 might be used to make differential diagnosis between COPD-PH and IPAH group.

Cav1 was highly expressed in vascular endothelial cells but less in smooth muscle cells. The expression of Cav1 was decreased in the plexiform lesion from IAPH patients' lung tissue [20]. The expression in the smooth muscle cell was increased and immunoblotting from whole lung prepared revealed decreased expression of Cav1 [25, 26]. In this study, we further demonstrate that the serum Cav1 level in IPAH patients was also decreased (Figure 2(a)), and the difference was significant between IPAH, COPD-PH, and normal subjects. By using serum Cav1 level 17.17 pg/mL as cutoff value in the diagnosis of IPAH, there were fare sensitivity (0.6) and good specificity (1.0) (Figure 3(a)).

In a small number of COPD with PH patients (mean PAP: 29.5 ± 5.1 mmHg), the intimal expression of Cav1 was decreased as compared with COPD patients without PAH (mean PAP: 16.7 ± 2.7 mmHg) [27]. Our data did not show significant difference between COPD-PH and normal subjects, but there was significant difference between COPD-PH and IPAH patients (Figure 2(a), *p* = 0.047). Although smooth muscle proliferation with increasing Cav1 expression was noted in both COPD-PH and IPAH patients, our results suggested the serum Cav1 level correlated with its expression in endothelial cells but not the smooth muscle cells. Our data suggest that Cav1 may be potential biomarkers for elevated PA pressure and could be used for differential diagnosis of COPD-PH and IPAH.

NT-proBNP is secreted by the ventricles of the heart in response to excessive stretching of cardiomyocytes. Serum NT-proBNP elevated in both left and right ventricle dysfunction [28, 29]. In COPD patients with PAH and right heart failure, the NT-proBNP was also elevated [30]. In our results (Figure 2(b)), NT-proBNP levels were significantly higher in both COPD-PAH and IPAH groups than normal subjects, but there was no significant difference between the disease subjects. hsCRP, a nonspecific biomarker in response to different pathogenesis of inflammation, was higher in COPD patients due to chronic lung inflammation (Figure 2(c), *p* = 0.017) than normal group. But there was no difference between COPD-PH and IPAH groups. Mutations in the BMPR2 gene resulted in the development of familial primary pulmonary hypertension, but the role BMPR2 mutations play in the development of PH has not been clarified. Cav1 and BMPR2 were colocalized in both endothelial and smooth muscle cell membrane [31, 32] and Cav1 was suggested to regulate BMPR2 downstream signaling. In this study (Figure 2(d)), serum BMPR2 level was not significantly different between normal subjects versus IPHA patients and normal subjects versus COPD-PAH patients. But the differences between IPAH and COPD with PAH were significantly different (*p* = 0.019). Further study may be indicated to elucidate the relation between Cav1 and BMPR2 in IPAH patients.

Taken together, our results demonstrated that reduced serum Cav1 level may be a potential biomarker in IPAH diagnosis and could be used for differential diagnosis of pulmonary artery hypertension patients between idiopathic pulmonary hypertension and COPD.

## 5. Study Limitation

This is a small number cross-sectional study. COPD-PH is more frequent in males; IPAH is more frequent in females. It is difficult to correct the match number of patients in gender. The IPAH patients included were at different treatment status, including newly diagnosed IPAH without medication to double or even triple drugs combined therapy. The functional status of heart failure may also influence Cav1 serum level. Therefore, we find only poor correlation between Cav1 level and pulmonary artery pressure, pulmonary vascular resistance, and 6-minute wall test. However, our results suggested serum Cav1 level might be used as an easy and convenient way for IAPH initial diagnosis. Future studies are necessary to include more patients at different stages of disease, to evaluate Cav1 level in response to different treatment, to predict the IPAH progression and long-term prognosis.

## Figures and Tables

**Table 1:** Demographic data patients with pulmonary artery hypertension and healthy controls.

	Non-PAH (*n* = 27)	COPD-PH (*n* = 20)	IPAH (*n* = 20)	*p* value
Age, yrs	51.30 ± 11.71 (64–38)	58.9 ± 12.96 (75–39)	45.4 ± 16.16 (78–18)	0.16
Sex (male/female)	22/5	16/4	6/14	0.000
Height, cm	161.94 ± 6.84 (182–152)	160.63 ± 7.22 (169.5–144)	158.93 ± 7.53 (176–147)	0.366
Weight, kg	66.65 ± 12.31 (86.5–49.5)	67.28 ± 12.50 (94–46)	63.51 ± 13.43 (98–46)	0.578
BMI, kg/m^2^	25.45 ± 3.91 (33.76–18.29)	16.16 ± 5.10 (38.58–17.10)	25.15 ± 5.64 (42.7–18.36)	0.791
History of DM	5 (18.5%)	5 (25.0%)	0 (0)	0.68
History of HTN	8 (29.6%)	10 (45.5%)	4 (18.2%)	0.117
PAP peak (mmHg)	17.69 ± 4.48 (8–25.8)	46.76 ± 12.74 (35–72.6)	96.37 ± 30.76 (47.30–169)	0.000
PAP mean (mmHg)	12.36 ± 2.89 (6.3–18.80)	31.14 ± 7.93 (23.4–48.4)	57.79 ± 14.87 (29.60–84.5)	0.000
SBP, mmHg	133.35 ± 16.01 (170–104)	132.80 ± 22.24 (176–96)	113.20 ± 23.10 (175–86)	0.002
DBP, mmHg	79.77 ± 9.60 (101–60)	81.80 ± 16.87 (119–62)	77.80 ± 16.87 (128–54)	0.683
TC, mg/dL	176.88 ± 35.35 (236–92)	183.35 ± 44.46 (281–105)	146.25 ± 33.39 (188–103)	0.078
HDL_C, mg/dL	41.6 ± 9.31 (59–29)	48.8 ± 38.5 (166–6)	52.4 ± 14.84 (66–33)	0.753
TG, mg/dL	142.88 ± 69.20 (373–53)	127.50 ± 94.72 (458–23)	79.88 ± 33.28 (148–38)	0.139
Creatinine, mg/dL	1.08 ± 0.27 (2–0.7)	1.44 ± 0.75 (4.4–0.8)	0.88 ± 1.6 (1.2–0.6)	0.515
AC_sugar, mg/dL	108.23 ± 26.36 (177–79)	110.50 ± 42.04 (235–49)	108.75 ± 34.78 (189–83)	0.980
Caveolin-1 pg/mL	173.57 ± 135.18 (47.22–409.44)	163.04 ± 146.59 (56.61–425.54)	33.81 ± 36.3 (18–235)	0.029
hsCRP mg/dL	0.18 ± 0.23 (0.1–0.95)	1.02 ± 1.30 (0.13–4.38)	0.37 ± 0.62 (0.03–2.75)	0.007
NT-proBNP pg/mL	59.83 ± 64.84 (4.0–336)	1426 ± 1231 (140–2790)	933.6 ± 891.3 (107–2120)	0.004

PAH: pulmonary artery hypertension, COPD-PH: chronic obstructive pulmonary disease with pulmonary hypertension, IPAH: idiopathic PAH, PAP: pulmonary artery pressure, SBP: systolic blood pressure, DBP: diastolic blood pressure, hsCRP: high-sensitivity C-reactive protein, and NT-proBNP: N-terminal of the prohormone brain natriuretic peptide.

**Table 2:** Sensitivity and specificity data for cutoff point of Cav1 and other biomarkers in IPAH patients.

Biomarker	Cutoff value	Sensitivity	Specificity
Cav1	17.17 pg/mL	0.588	1
NT-proBNP	89.25 pg/mL	0.889	0.778
hsCRP	0.27 mg/dL	0.389	0.852
BMPR2	3.71 pg/mL	1	0.429
